# Does Dementia Have a Microbial Cause?

**DOI:** 10.3390/neurosci3020019

**Published:** 2022-05-17

**Authors:** Remi L. Landry, Monica E. Embers

**Affiliations:** 1 Department of Tropical Medicine, Tulane University School of Public Health and Tropical Medicine, New Orleans, LA 70112, USA; rlandry10@tulane.edu; 2 Division of Immunology, Tulane National Primate Research Center, Tulane University Health Sciences, Covington, LA 70433, USA

**Keywords:** dementia, amyloid, *Borrelia*, HSV-1, Alzheimer’s, neurodegenerative

## Abstract

The potential contribution of pathogenic microbes to dementia-inducing disease is a subject of considerable importance. Alzheimer’s disease (AD) is a neurocognitive disease that slowly destroys brain function, leading to cognitive decline and behavioral and psychiatric disorders. The histopathology of AD is associated with neuronal loss and progressive synaptic dysfunction, accompanied by the deposition of amyloid-β (Aβ) peptide in the form of parenchymal plaques and abnormal aggregated tau protein in the form of neurofibrillary tangles. Observational, epidemiological, experimental, and pathological studies have generated evidence for the complexity and possible polymicrobial causality in dementia-inducing diseases. The AD pathogen hypothesis states that pathogens and microbes act as triggers, interacting with genetic factors to initiate the accumulation of Aβ, hyperphosphorylated tau protein (p-tau), and inflammation in the brain. Evidence indicates that *Borrelia* sp., HSV-1, VZV (HHV-2), HHV-6/7, oral pathogens, *Chlamydophila pneumoniae*, and *Candida albicans* can infect the central nervous system (CNS), evade the immune system, and consequently prevail in the AD brain. Researchers have made significant progress in understanding the multifactorial and overlapping factors that are thought to take part in the etiopathogenesis of dementia; however, the cause of AD remains unclear.

## 1. Introduction

**Figure 1:**
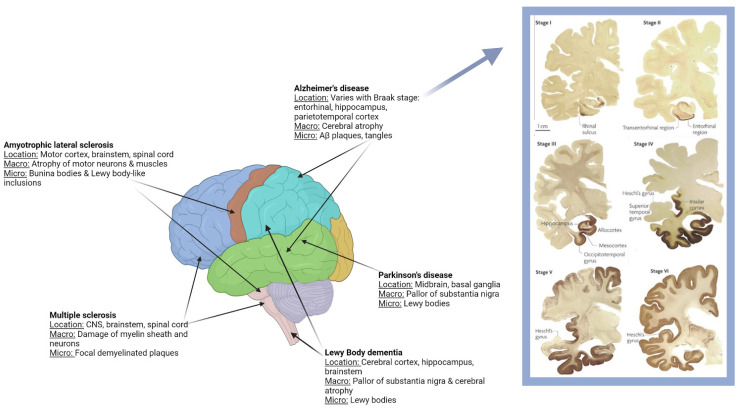
Association of specific diseases with affected regions of the brain. All figures/illustrations were created with BioRender.

Neurodegenerative diseases are gaining increased attention as they represent a major public health problem with a substantial socioeconomic impact, affecting more than 50 million Americans each year. As populations of older Americans grow rapidly and live longer due to advances in social and environmental conditions, so too will the number of people living with neurodegenerative disorders. Neurodegenerative disorders are defined as sporadic, age-related, and hereditary conditions characterized by cognitive decline [1]. Many factors have been associated with neurodegenerative disease, including genetics, nutritional deficiencies, environmental factors, infectious agents, and certain metals [2]. These factors and others can cause alterations in brain biochemistry and signal transmissions in nerve cells, which can result in neuronal degeneration, cell dysfunction, and neural death, leading to neurological disorders and dementias. The most problematic neurodegenerative diseases include Alzheimer’s disease (AD), Lewy body dementias, amyotrophic lateral sclerosis (ALS), Parkinson’s disease (PD), multiple sclerosis (MS), prion diseases, and other dementias. The clinical manifestations of a particular neurodegenerative disease reflect the type of pathological protein that accumulates, the regions of the brain impacted, and the types of neurons that are susceptible to cytotoxic events (Figure 1). Although traditionally neurodegenerative disorders are clinicopathologically distinct entities, it is increasingly recognized that the clinical presentations of disorders can overlap. Considerable clinical and neuropathological overlap of two or more disorders is not uncommon. For instance, patients with frontotemporal dementia (FTD) may present with ALS, patients with FTD may develop symptoms resembling PD, and many patients with Lewy body dementia also have overlapping AD [3]. AD pathology is further delineated by Braak stage, as multiple regions are sequentially involved (Figure 1 [4]).

Dementia has become a serious health problem in the industrialized world [5]. A large cohort of the American population—the baby-boom generation—has started reaching age 65 and beyond, the age range when the risk for Alzheimer’s and related dementia is greatest [6]. In 2021, the population of Americans aged 65 and older was an estimated 58 million, but this number is expected to grow to 88 million by 2050 [7]. Consequently, the prevalence of AD in the number of those 65 and older is expected to increase from a current estimate of 6.2 million people to 13.8 million by 2060 [5]. AD is officially listed as the sixth leading cause of death in the U.S. and the fifth leading cause of death for those aged 65 and older [8].

Alzheimer’s disease and related dementia results from a chronic inflammatory neurodegenerative disease process that slowly destroys brain function leading to cognitive decline, behavioral and psychiatric disorders, and declines in daily life functions. Most commonly, AD is manifested as a progressive amnesia-predominant multidomain cognitive impairment [9]. Typically, a gradually degenerating memory is the earliest cognitive deficit. In most cases, the ability to communicate and perform activities of daily living are severely impaired [10]. Other deficits, such as behavioral disorders, personality changes, visuospatial disturbances, and motor and sensory deficits, manifest in the advanced stages or atypical presentations of the disease [11,12,13,14]. Atypical presentations, including posterior cortical atrophy (PCA), primary progressive aphasia (PPA), and frontal variant AD, pose a diagnostic challenge for researchers and clinicians [9,15]. These atypical forms of AD account for 5% of AD patients at least 65 years old and account for roughly one third of AD patients below the age of 65 years [14,16]. The objective of this review is to consider the evidence for the role of microbes, and possible mechanisms of pathogenesis, in the development of Alzheimer’s and related dementias.

### 1.1. Clinical and Pathological Characteristics

Currently, the provisional diagnosis of AD is largely one of exclusion. Clinical history, psychiatric and neurological examination, cognitive testing, and neuroimaging all serve to exclude other common neurodegenerative disorders. The last 20 years have seen an expansion in research on biomarkers for AD. The core cerebrospinal fluid (CSF) biomarkers, total tau (T-tau), phosphorylated tau (p-tau), and amyloid-β (Aβ 42), have shown consistent high diagnostic accuracy for AD dementia [17]. Positron emission tomography (PET) imaging with amyloid-beta (Aβ) tracers and 2-[18F] fluoro-2-Deoxy-D-glucose (FDG) is a promising tool for the early detection of AD [18]. FDG-PET for AD pathology has become a prime modality for detecting brain changes in vivo in preclinical and early AD [18]. A definitive diagnosis, which is also regarded as the gold standard, requires a clinical assessment of probable AD followed by a histopathological examination of either biopsy or postmortem brain tissue for the presence of neurofibrillary tangles and amyloid plaques. The diagnosis of AD is further complicated by the similar clinical symptomatology of other neurodegenerative diseases.

Alzheimer’s disease is classified into two distinct forms: early-onset AD (EOAD) or sporadic late-onset AD (LOAD) [19]. Progress has been made with regard to understanding the pathological entities that arise in the AD brain, both for the early-onset and late-onset forms [20]. Both EOAD and LOAD have common characteristics, including oxidative stress, upregulated pro-inflammatory signaling, the accumulation of lesions and plaques, synaptic signaling deficits, atrophy, altered gene expression, and cognitive impairment.

Lewy body dementias include dementia with Lewy bodies (DLB) and Parkinson disease dementia (PDD). DLB and PDD are clinically similar diseases that share characteristic neuropathologic changes, including the deposition of α-synuclein in Lewy bodies and the loss of tegmental dopamine cell populations and basal forebrain cholinergic populations; these often exist coincident with AD pathology [21]. The clinical manifestations of DLB and PDD include the progressive cognitive impairment associated with parkinsonism, visual hallucinations, delusions, and fluctuations of attention and wakefulness [22]. While AD affects the brain’s ability to store new information in the form of memories, DLB targets a different set of cognitive functions, including problem-solving and reasoning. In general, women have a higher chance of developing AD, while men are at a higher risk for DLB. Often, patients with DLB and PDD have difficulty walking and struggle with balance, whereas physical deterioration does not usually occur in AD. Clinical differentiation between DLB and PDD is based on a distinction between the time of onset of parkinsonism and cognitive symptoms [22].

Early-onset, generally before the age of 65, is a rare familial form of AD that accounts for approximately < 5% of all cases of the disease [23]. Current estimates do not include people with Down syndrome (DS), but EOAD should comprise both Down-syndrome-associated AD (DSAD) and autosomal dominant AD (ADAD) [24]. However, it should be noted that not all EOAD are ADAD or DSAD. Genome-wide association studies (GWAS) have linked various genes that, when combined with other genetic and environmental factors, lead to AD. Although genetic variations are single-nucleotide polymorphisms (SNPs), a variation in a single base of the genome sequence among individuals, these genes might code for proteins that interact in various processes, leading to the development of AD [25]. To date, 106 mutations on three separate genes are associated with the early-onset form of AD: the amyloid precursor protein (APP) gene on chromosome 21, the presenilin 1 (*PSEN1*) gene on chromosome 14, and the presenilin 2 (*PSEN2*) gene on chromosome 1 [26,27,28,29]. *PSEN1*, the most frequently mutated gene, accounts for the majority of early onset (prior to age 50) AD cases [30]. These AD-causing mutations are characterized by primary alterations in amyloid metabolism, leading to an overabundance in the Aβ_42_ species, neuronal cell death, and dementia [30]. APP is pathological with respect to dementia when it is duplicated (duAPP). It has been recognized that triplication of APP in patients with DS leads to Alzheimer’s symptoms early in life by the over-expression of APP, followed by neurodegeneration and Aβ deposition [31,32,33]. Interestingly, non-DS people who inherit triplication of the APP gene will develop AD symptoms by age 60 with complete penetrance [34].

The late-onset form of the disease is defined as AD with onset at age 65 years or older and accounts for the majority of AD cases. A far more common genetic risk factor for late-onset AD was identified as the type ε4 allele of the gene for apolipoprotein E (APOE) [35,36,37]. Located on chromosome 19, the APOE ε4 allele has essential roles in cholesterol homeostasis and triglyceride metabolism. Unlike the genes associated with the early-onset form, the APOE 4 allele is not deterministic or sufficient to cause AD but rather confers a higher risk for developing LOAD [36,38]. Compared with non-carriers, individuals with one APOE ε4 allele have a three- to four-fold risk of developing AD, while those with two APOE ε4 alleles have a 5–18 times greater risk of developing the disease [27]. Approximately 50–60% of AD cases are attributable to the APOE ε4 gene, and it is estimated that up to 25% of the U.S. population are carriers for the gene [27]. In terms of the cellular and biochemical processes that result in the clinical manifestations, aggregating Aβ and p-tau can cause oxidative stress, the dysregulation of Ca^2+^ homeostasis, mitochondrial dysfunction, synapse degeneration, and eventually, neuronal death [39]. As there is no known cure for AD and current medical treatment is not optimal, it is necessary to identify preventive strategies that can delay the onset or reduce the risk of AD. The major hurdle in understanding AD is the lack of comprehensive knowledge about its etiology and pathogenesis.

### 1.2. Pathogenesis of Alzheimer’s Disease and Associated Dementias

**Figure 2:**
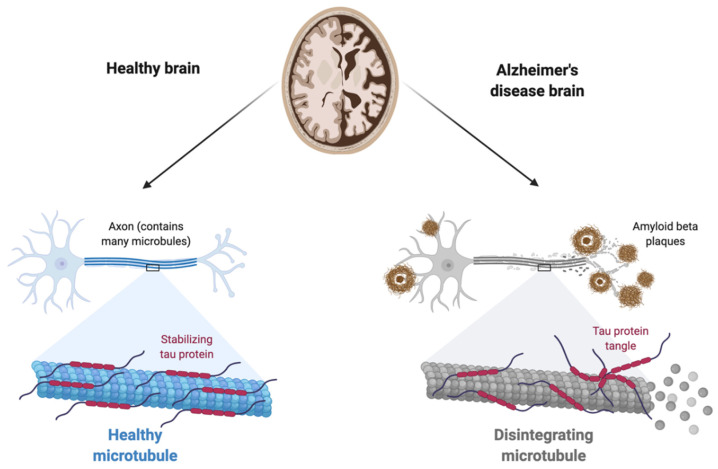
Differences between a healthy brain and an Alzheimer’s disease brain.

Since 1907, when Aloysius Alzheimer first described AD, senile plaques and neurofibrillary tangles have become the hallmark neuropathological features of AD [40,41]. The histopathology of AD is associated with neuronal loss, the deposition of Aβ peptide in the form of neuritic senile plaques (NSPs), abnormal aggregated tau protein (p-tau) in the form of neurofibrillary tangles (NFTs), neuropil threads, and often deposits of cerebrovascular amyloid [42] (Figure 2). Several hypotheses have been put forward to explain the pathogenic process in Alzheimer’s disease; these include the amyloid, inflammatory, and microbial hypotheses [41,43,44].

#### 1.2.1. The Amyloid Hypothesis

Numerous large-scale studies have examined the relationship between various AD biomarkers and cognitive decline. Investigators refined the ability to detect and measure CSF levels of tau protein and Aβ that were indicative of AD pathology in the brain [37]. Gene mutations have been shown to alter APP metabolism, facilitating the production of aggregation-prone Aβ species [45]. Such findings are the basis for the broadly accepted amyloid cascade hypothesis. This hypothesis states that the accumulation of misfolded tau proteins and amyloid plaques constitute synaptotoxicity and neurofibrillary tangles, which result in the hallmarks of AD pathophysiology, neuronal injury, and neurodegeneration [37]. This hypothesis has been influential in the development of treatment strategies for AD, as it posits that amyloid can be cleared and the cascade of events that lead to dementia can be prevented [46]. However, there is increasing evidence that raises doubt about the amyloid cascade hypothesis. Studies have shown that neurodegeneration, marked by an increase in p-tau in cerebrospinal fluid, can occur before amyloidosis and is predictive of cognitive decline in AD [47]. Around 400 clinical drug trials based on the amyloid cascade hypothesis have failed, leading researchers to search for a reliable explanation. It is important to point out the long duration of the preclinical stage of AD, which may imply that amyloid-targeting drugs could have some prophylactic uses. Although the amyloid cascade hypothesis may likely be true for the familial form of the disease, evidence suggests the mechanisms underlying LOAD could be different [48].

#### 1.2.2. The Inflammatory Hypothesis

Chronic pro-inflammatory immune activity is progressively being recognized as a crucial component of neurodegenerative disorders, including all types of dementia. Several lines of evidence have implicated inflammatory mediators and the innate immune system of the brain in the pathological etiopathogenesis of late-onset AD. Despite different etiologies, common features of neurodegenerative disorders are chronic activation of the innate immune system and the influx of immune cells across the blood–brain barrier (BBB), which controls the passage of molecules into and out of the brain [49]. Dysfunction of the BBB, activated endothelial cells, and a decrease in tight junction proteins occurs in Alzheimer’s disease. A central tenet of the hypothesis proposed by Krstic and Knuese is that inflammation becomes self-perpetuating, contributes to brain pathology, and induces neurodegeneration and cognitive dysfunction as well as the dysfunction of synapses [48]. The inflammatory process is mediated by activated microglia, which respond to neuronal damage by phagocytizing the damaged cells. Microglia, however, become less efficient in this process as we age. If the inflammatory response is uncontrolled, chronic systemic inflammation induces a ‘priming’ of microglial cells and extensive astrogliosis [50]. These activated microglia release proinflammatory cytokines, reactive oxygen intermediates, proteinases, and complement proteins. Cytotoxic molecules, such as IL-6, IL-1β, IL-18, TNF-α, and IFN-γ, released from the activated microglia cause neuronal injury.

An infection in the brain not only results in the release of cytotoxic molecules, but it also stimulates various classes of T cells [51]. Mucosal-associated invariant T (MAIT) cells are innate-like T cells that recognize microbial vitamin-B-derived metabolites [52,53,54,55,56]. These metabolites are presented by MR1, a major histocompatibility complex (MHC) class Ib molecule [56]. Under healthy conditions, MR1 is expressed at low levels on the cell surface. However, in a diseased state, it is upregulated [54,56,57,58,59,60]. When MAIT cells recognize MR1 on antigen-presenting cells, they become activated and subsequently secrete proinflammatory cytokines and cytotoxic molecules [52,61,62,63]. Increasing evidence has highlighted the role of chronic MAIT cell activation in neurodegenerative disorders. In a number of CNS diseases, MAIT cells infiltrate CNS lesions and secrete high amounts of proinflammatory cytokines, resulting in inflammation and a poor outcome of the disease [64,65]. When neuroinflammation from CNS disorders causes significant damage to the brain, microbes enter the CNS and have the ability to activate the MR1/MAIT cell axis, possibly leading to further neuroinflammation.

Chronic inflammation impairs the mechanism for clearing abnormal proteins in aging brains, which leads to the formation of paired helical filaments, the accumulation of APP, and synaptic dysfunction, ultimately resulting in tissue degeneration and the development of CNS disorders [1]. Increased Aβ accumulation in synapses leads to reduced synaptic activity and consequent neuronal damage and signal dysfunction. Synaptic failure is an early event in AD and is thought to be linked to APP [66]. The role of inflammation is multifold: (1) it can be continually perpetuated, stimulating brain cells, and (2) it activates pathways that lead to AD-specific pathology, synaptic and cognitive dysfunction, neurodegeneration, and blood–brain barrier (BBB) permeability. However, the precise role of inflammation in disease pathophysiology is a controversial one, ranging from a potential cause of disease to a by-product of disease.

#### 1.2.3. The Microbial Hypothesis

An increasing number of experimental and epidemiologic studies have implicated various bacterial, viral, parasitic, and fungal agents as risk factors for neurodegenerative diseases and dementias. Miklossy, and MacDonald and Miranda wrote some of the first papers implicating microbes, particularly spirochetes, in AD pathogenesis [67,68]. This hypothesis is closely connected to the inflammatory hypothesis, as microbes are thought to trigger Alzheimer’s pathology through inflammatory mechanisms. While the CNS is protected by the BBB, a broad spectrum of pathogens can gain access and cause illness [69]. As pathogens replicate, they release pathogen-associated molecular patterns (PAMPs) that can be identified by pattern recognition receptors (PRRs) expressed on antigen-presenting cells [1,49]. Infectious factors can activate the glial cells that produce inflammatory molecules, which in turn leads to the exacerbation of other dementia pathologies. Pathological Aβ deposits and damage to the CNS during infection activate glial cells, lymphocytes, and macrophages and trigger the release of inflammatory mediators to eliminate the invasive pathogens. However, if the activity of stimulating factors and the inflammation processes are dysregulated, the body switches from an acute to a chronic inflammatory response [70,71]. Aβ peptide is known to participate in the innate immune response and has been found to have antimicrobial activity, suggesting that microbial infections induce the formation of Aβ-containing senile plaques as a protective mechanism [22]. Thus, the importance of inflammatory processes in the pathogenesis of AD and related dementia has generated overwhelming evidence for the complexity and possible polymicrobial causality of dementia.

#### 1.2.4. Role of the Gut–Brain Axis (GBA)

Bridging these three theories is the possible contribution of the gut microbiota and its dysbiosis in the mechanisms leading to neurological damage [72]. In particular, the permeability of the blood–brain barrier is known to be affected by changes in the composition of the gut microbiota through the GBA [73], a neural, immune, endocrine, and metabolic connection between the brain and the intestinal tract. Bacteria in the gut may secrete lipopolysaccharides and amyloid protein, which may contribute to AD pathogenesis. Finally, gut dysbiosis can induce inflammatory responses, including the inflammasome pathway, which have been linked to neurotoxicity.

## 2. Discussion: The Microbial Hypothesis

**Figure 3:**
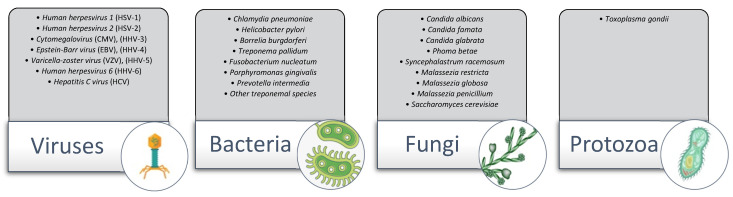
Pathogens associated with the development of AD.

Genetic, biochemical, and immunological analyses have provided a relatively detailed knowledge of the pathology of AD, but the understanding of the trigger events leading to the many cascades resulting in neurodegeneration is still limited. Hence, the etiology of dementias has remained elusive. However, a number of recent and ongoing epidemiological and experimental studies have implicated microbial infections in the pathogenesis of late-onset Alzheimer’s disease. Pathogens can produce slow-progressing chronic diseases. Infectious agents have been linked to stomach ulcers, atherosclerosis, cardiovascular and cerebrovascular disorders, lung disease, inflammatory bowel diseases, and neuropsychiatric disorders. The AD pathogen hypothesis states that microbes and pathogens act as triggers, interacting with genetic factors to initiate the accumulation of Aβ, p-tau, and inflammation in the brain [74]. Since the 1970s, the microbial hypothesis has gradually gained popularity, despite denial of the idea that a microbial infection might be a trigger event to AD and other dementias. Many attempts have been made to identify infectious agents responsible for Alzheimer’s and related dementias. In recent years, evidence suggests that herpes simplex virus type 1, *Chlamydophila pneumoniae*, *Candida albicans*, spirochetes, periodontal pathogens, and *Helicobacter pylori* can infect and evade the immune system, increase the inflammatory states, and consequently prevail in the AD brain. Pathogens possibly associated with the development of AD are presented in Figure 3. None of the agents studied have been universally accepted to be etiologic for dementia-related neuropathology. However, this review will focus on the contribution given to herpes simplex virus type-1, treponemal species, *Borrelia burgdorferi*, *Chlamydia pneumoniae*, and *Candida albicans*.

### 2.1. The Herpesviridae Family

There is considerable evidence implicating herpesviruses in the pathogenesis of Alzheimer’s disease. Herpes simplex virus (HSV) type 1 and type 2 are ubiquitous pathogens that persist for the life of the infected individual. Often, only a certain population of those infected actually show symptoms, while the remainder are asymptomatic [75]. Herpes simplex virus type 1 (HSV-1) affects the majority of the population, attaining 90% prevalence by the sixth decade of life, with geographic variations. During 2015–2016, in the US alone, the seroprevalence of HSV-1 was 47.8% and the seroprevalence of HSV-2 was 11.9% in the National Health and Nutrition Examination Survey [76]. While there are eight types of herpesviruses belonging to three subclasses, only seven are implicated in Alzheimer’s disease: HSV-1, HSV-2, VZV, human herpesvirus 6A, human herpesvirus 7, CMV, and EBV.

The double-stranded neurotropic virus can establish lifelong latency in nervous tissues, particularly the trigeminal ganglia of the peripheral nervous system, and can reactivate periodically in response to a variety of stimuli, such as immunosuppression, inflammation, peripheral infection, or other stressors [77]. In 1982, Melvin Ball hypothesized that HSV-1 was causative in AD pathogenesis [78]. The viral concept of AD proposes that latent HSV-1 located in the trigeminal nerve could reactivate and ascend into areas of the brain. The effect of repeated reactivation, inflammation, and direct viral action may be cumulative and can lead to the development of AD. Herpes simplex encephalitis (HSE) affects the frontal lobes, temporal lobes, and hippocampus [74]. Interestingly, herpes encephalitis shows cognitive, memory, and behavioral declines analogous to those seen in AD. HSE is frequently associated with epilepsy, which is recognized as a comorbidity in early onset AD patients. Conversely, with APOE4 there is higher epilepsy risk [79,80]. Thus, there are functional links between HSE and AD, AD and epilepsy, and consequently, evidence linking HSV-1 to AD.

Work from many laboratories strongly supports the concept that HSV-1 enters the brain in older age as the immune system declines. Dr. Itzhaki was a strong proponent for the role of viruses in Alzheimer’s disease. She proposed that they can be reactivated periodically, subsequently inducing brain inflammation and pathology. This hypothesis is supported by multiple lines of evidence. In 1991, Jamieson and colleagues discovered that HSV-1 DNA is present in a high proportion of both elderly normal subjects and AD patients, where the former were infected but asymptomatic [81]. In addition, the virus might be a risk factor for AD when it occurs simultaneously with APOE-ε4 [82,83]. The APOE-ε4 allele is a well-established genetic risk factor for AD but has also been shown to influence susceptibility to viral infections [84]. It was hypothesized that the damage caused by HSV-1 depends on APOE-ε4 carriage, i.e., APOE-ε4 carriers suffer either great viral damage upon reactivation or have poorer repair of such damage [82]. A concurrent finding was that APOE-ε4 is a risk for herpes labialis (cold sores), which is usually caused by the virus in the peripheral nervous system [82]. In genital herpes, caused by HSV-2, APOE-ε4 is a risk for the recurrence of genital ulcers [85]. Additionally, studies on the distribution of HSV-1 DNA in humans revealed that viral DNA was found within senile plaques from the temporal and frontal cortices of AD sufferers and was present with a high frequency in elderly brains, in contrast to its presence in the brains of young people [86]. Ninety percent of plaques contained HSV-1 DNA, and in AD brains, 72% of viral DNA was associated with plaques compared to 24% in healthy elderly brains [86]. This perhaps reflects the reduced Aβ production or greater clearance in healthy individuals. It has been suggested that the virus can easily enter the brain due to the higher permeability of the BBB and impaired immune response in elderly individuals [1]. These findings support that the virus causes the formation of toxic Aβ plaques and, therefore, establishes a causal role for HSV-1 in AD pathogenesis. It is worth stressing that the evidence for a role of HSV-1 in AD does not preclude a role for other microbes that are implicated in AD. One or more microbes might be involved in the development of disease in AD patients whose illness is not accounted for by HSV-1 (in combination with APOE-ε4).

Clinical epidemiological studies also support the viral hypothesis of AD. A large prospective population-based study showed an increased risk of AD in elderly subjects with positive titers of anti-HSV-1 IgM, a marker for of primary or reactivated infection [87]. Letenneur et al., however, did not see an increased risk with anti-HSV-1 IgG antibodies, markers of life-long infection [88]. Lövheim et al. also showed that positivity for herpes simplex virus IgM was found to increase the risk of developing AD by almost two-fold, whereas the presence of IgG did not affect the risk [89]. The Itzhaki lab also detected intrathecal antibodies to HSV-1 in the CSF of a high proportion of both healthy elderly people and AD patients [90]. This finding indicated that HSV-1 could actively replicate in the brain, causing direct damage or the induction of inflammatory processes. It was noted that inflammation caused by HSV-1 corresponded to areas of damaged brain tissue in early stages of the disease [91]. Subsequent studies have revealed that HSV-1 causes Aβ deposition and AD-like p-tau in infected cell cultures and in mice [86].

Recently, studies conducted in a large Taiwanese population yielded results that link antiherpetic medication with a decreased incidence of dementia [92]. The authors investigated a cohort of roughly 33,000 subjects aged ≥50 years old during the year 2000, who were diagnosed with HSV-1 or HSV-2 infections. The incidence of dementia in the age- and gender-matched control group and the HSV group were investigated for 10 years (2001–2010). The risk of developing dementia in the HSV group was 2.56-fold greater; the main effect was seen in those with HSV-1 infections. Interestingly, a group of HSV-infected patients who had been treated with one of various antiherpetic agents showed a reduction in the incidence of dementia compared to those who received no treatment [75]. These studies postulate that antiherpetic medications are likely associated with decreased risk of developing dementia and could be used to prevent or slow disease progression. While these studies are promising, there are no data on the effect of antivirals in subjects who are already suffering from AD. Along with the data on HSV-1 presence in elderly brains and its link to APOE-ε4 in AD, these studies support a causal role of HSV-1 in AD [81,90].

**Figure 4:**
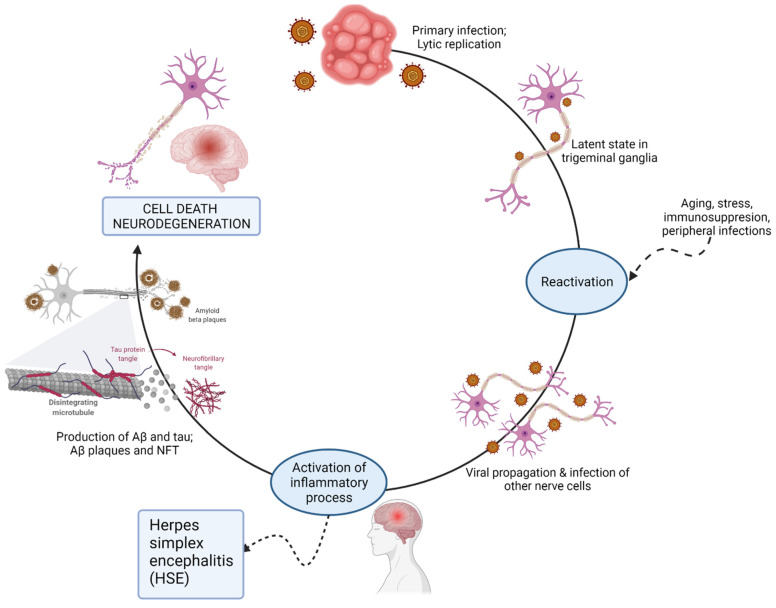
Possible mechanisms for virus-induced neurodegeneration.

It is also worth noting that other viruses in the Herpesviridae family, such as HHV-6/7, HHV-2, varicella zoster, and cytomegalovirus, can establish latency and persist for life after the initial infection [91]. However, studies focused on the Herpesviridae family in the context of age-related diseases are limited. In a recent study, Readhead et al. suggested that HHV-6A and HHV-7 could be causal contributors to AD [93]. HHV-6A and HHV-7 could induce cell apoptosis in developing thymocytes following primary infection [94]. The activation of HHV-6A and HHV-7 lytic replication in infected neurons may establish the process by which the viruses cause brain damage [95]. It has been previously reported that natural killer (NK) cells are activated in response to infection. Thus, indirect effects of the activated host immune system could also destroy brain tissue. Some have theorized that the APP pathway might be hijacked by HHV-6A/7 in the aging brain to cause damage [95]. The viral induction of AD is depicted in Figure 4.

### 2.2. Oral Bacteria

Periodontal disease is an inflammatory dysbiosis with a significant bacterial burden that elicits a systemic inflammatory response and the release of proinflammatory cytokines. There are numerous oral pathogenic bacteria, several of which are invasive, including, but not limited to, *Treponema denticola* and *Porphyromonas gingivalis*. *Treponema denticola* (along with several other oral treponeme species, such as *T. pectinovorum* and *T. socranskii*) can cause periodontal disease, which, if untreated, can result in edentulism, commonly known as tooth loss. According to the World Health Organization, an estimated 5–20% of adults 65 and older suffer from chronic periodontitis [96]. Chronic periodontitis has been implicated in various systemic conditions, including rheumatoid arthritis, diabetes mellitus, and more recently, AD. Diseased periodontal pockets host roughly 200 to 300 bacterial species [97]. The ulcerated lining of the periodontal pockets can reach 20 cm^2^, providing bacteria with direct access to the systemic vasculature [98]. Following entry into circulation, bacterial and inflammatory molecules can access the brain.

There are currently two proposed pathways by which these invasive periodontal spirochetes can reach the brain: inflammatory and bacterial. It has been proposed that chronic periodontitis leads to tissue destruction by inducing an immune response. The interaction between periodontopathic bacteria and host immune cells results in a locally increased production of IL-1β, IL-6, IL-8, and TNF-α. Prolonged exposure of the brain to spirochetal infection and inflammatory mediators “primes” microglia in individuals and results in an inadequate neutralization of invading pathogens reaching the brain. Pro-inflammatory mediators penetrate the BBB, act on the already-primed microglial cells, and trigger the production of Aβ and tau phosphorylation. The resulting neuronal damage could proceed to AD development. Inflammation plays an essential role in periodontitis and, accordingly, has the potential to be causal in AD [99]. The second mechanism by which the bacteria could contribute to brain inflammation is through bacterial products [98]. Once in the brain, periodontopathic bacteria that are rich in lipopolysaccharide (LPS) or their products are capable of stimulating cytokine production. Chronic neuronal stimulation by LPS may result in damage to neurons and induce astrocyte activation and glia [100]. Systemic lipopolysaccharides can quickly increase cytokines within the hippocampus. In the brain, these bacteria and inflammatory molecules can enhance AD-specific pathology, subsequently resulting in neurodegeneration.

Researchers have also found conflicting evidence regarding the association between *Treponemal* spp. and cognitive decline. In summary, 14 longitudinal studies using different designs, definitions, and confounders were conducted in Asia, Europe, and the US [44,101,102,103,104,105,106,107,108,109,110,111,112,113,114,115]. A positive association was found in 71% of these studies. Considering studies with at least 10 years of follow up, six out of eight found a positive association. In a cohort of 60 elderly subjects with Alzheimer’s disease, periodontal disease exposure was associated with an increase in cognitive decline over a 6-month follow-up [115]. However, in a prospective cohort of over 550 adults aged 52–75 years, those with periodontal disease did not decline faster compared to the unaffected controls [103]. A case-control study revealed those with periodontal disease were more likely to have cognitive impairment, while a nested case-control study showed there was no association [116,117].

**Figure 5:**
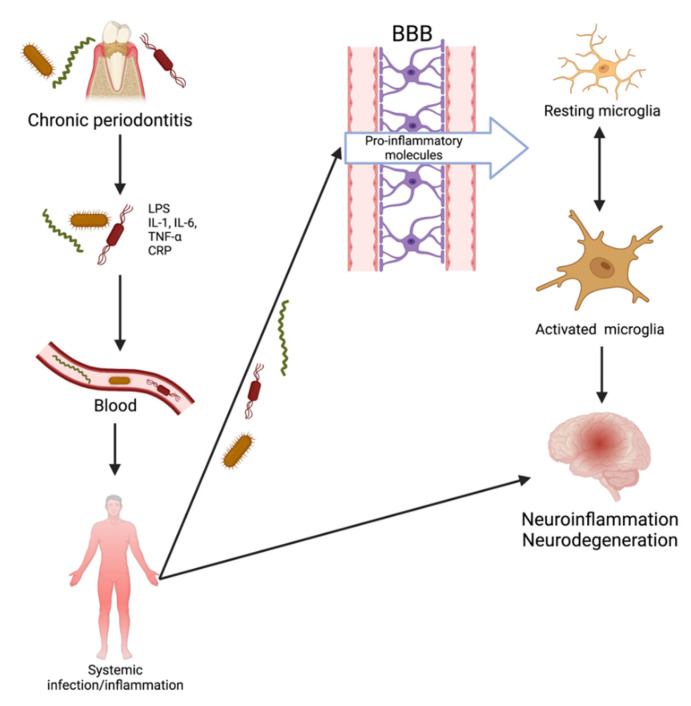
Transition of periodontitis to neurological disease.

Mounting evidence exists for the role of *P. gingivalis* in AD, resulting from animal, in vitro, and clinical studies [118,119,120,121,122,123,124,125,126,127]. Almost all studies have shown neuroinflammation in the brains of experimental animals, but four confirmed the presence of the pathogen DNA and lipopolysaccharides [119,120,123,127] in the CNS. Similarly, two studies showed neurodegeneration, and four showed cognitive impairment in animal models [119,121,122,124,126]. Recently, Ilievski et al. confirmed these results with a periodontal model by showing that *P. gingivalis* reached the brain and induced neuroinflammation, AD-specific pathology, and neurodegeneration [120]. Dominy et al. found that when added peripherally, inhibitors of gingipains (trypsin-like cysteine proteinases that can degrade cytokines) were able to counteract the pathological effects of *P. gingivalis* in the brain [119]. These studies show that the periodontal bacterial species are able to induce brain pathology, cognitive impairment, neurodegeneration, blood–brain barrier disruption, and neuroinflammation (Figure 5). Altogether, these various studies provide support for the theory that periodontitis can occur before AD pathology and that it has the potential to induce Alzheimer’s disease pathophysiology.

### 2.3. Borrelia burgdorferi

Spirochetes are corkscrew-shaped bacteria that are the causative agents for several chronic diseases including syphilis, Lyme disease, and periodontal disease. Because spirochetes are strongly neurotropic, there is an increasing amount of data that indicate that spirochetal infection causes Aβ deposition and plaque- and tangle-like lesions and, therefore, might be involved in dementia and AD pathogenesis. Spirochetes disseminate as individual bacteria to the cerebral cortex and may form plaques. Spirochetes evade host defenses and sustain chronic infection and inflammation, which may explain the slowly progressive course of dementia in AD [128]. The spirochetal aggregates appear similar in morphology to cortical senile plaques in AD [129]. Histochemical, immunohistochemical, and in situ hybridization techniques further demonstrated that Aβ and bacterial DNA are vital components of both pure spirochetal aggregates and senile plaques [128]. Researchers have reported that the number of spirochetes and plaques in the hippocampus and frontal cortex increases with the severity of cortical atrophy [130]. Previously, researchers have suggested that APP is an integral part of spirochetes, indicating that bacterial amyloid could contribute to senile plaque formation [67].

One spirochete that has been implicated in the etiology of Alzheimer’s disease is *Borrelia burgdorferi*. *B. burgdorferi,* a bacterium transmitted to humans through the bite of an *Ixodes* tick, is the etiologic agent of Lyme disease (LD). Lyme disease is characterized by erythema migrans, extreme fatigue, myocarditis, oligoarthritis of joints, and neurologic dysfunction. The most dangerous outcome of *B. burgdorferi* infection is associated with an invasion of the nervous system by the spirochete. Each year in the United States >30,000 cases are reported to the Centers for Disease Control and Prevention (CDC), but a recent estimate using other methods suggests the number of infections is closer to 476,000/year [131,132]. The organ pleiotropism of *B. burgdorferi* results in diverse manifestations, including neuroborreliosis [133], which occurs in approximately 10–15% of patients. CNS infection resulting in neurologic Lyme disease is well-documented [134]. The treatment of LD with oral or intravenous (for late stage) antibiotic therapy, in a majority of cases, leads to an improvement in symptoms after several weeks or months. However, a proportion of patients continue to experience symptoms, a phenomenon known as post-treatment Lyme disease (PTLD), which is considered by some to be a psychosomatic neurocognitive impairment [135]. However, animal studies have clearly demonstrated the persistence of the LD spirochete in multiple organs, including the brain, after antibiotic treatment [136,137,138]. Prior studies of PTLD patients showed immune activation in both CSF and serum. The activation of the inflammatory response in Lyme neuroborreliosis contributes to the pathogenesis of a broad spectrum of neurologic disorders. The clinical manifestations of neuroborreliosis may include ataxia, Parkinson-like symptoms, cognitive impairment, mental health disorders, paraparesis, and confusion. The discordant findings regarding the successful detection of the spirochetes involved in affected tissues has resulted in a stark difference of opinions. However, given the neurotropic effects of LD, many researchers have speculated a causal association between Lyme disease and neurodegenerative disorders.

While many researchers remain skeptical, there is strong evidence that shows *B. burgdorferi* can evade the host immune reactions and cause chronic infection, which further develops into neurological damage. The resultant inflammatory state leads to abnormal tau phosphorylation, microtubular dysfunction, and neurofibrillary tangle generation [139]. The pathogenic bacteria *Borrelia* have been shown to increase the permeability of the blood–brain barrier for entry, causing pleocytosis in the CSF, with white blood cell migration increasing as well [139]. Spirochetes then disseminate as individual bacteria to the cerebral cortex, and they are thought to form masses or plaques. *B. burgdorferi* spirochetes have been shown to produce amyloid deposits and tau hyperphosphorylation, indicating that bacteria and/or their degradation products may enhance the cascade of events leading to dementia.

In 1987, MacDonald and Miranda first reported the incidence of *B. burgdorferi* in the brains of AD cases [68]. The identification was validated using serological methods and morphological and immunohistochemical features. Several other researchers have reported similar findings. Fallon and Nields revealed the association of dementia and microgliosis with cortical atrophy in LD [140]. Meer-Scherrer et al. detected an association between *B. burgdorferi* DNA and neuropathology in the post-mortem brain tissue of a PTLD patient [140], and Miklossy detected spirochetes in the blood, CSF, and brain tissue of AD cases [141]. In addition, Miklossy and colleagues discovered that in AD patients NFTs were co-localized with Aβ plaques, and both contained *B. burgdorferi*-specific DNA [128]. Miklossy then examined 147 AD patients for the isolation of spirochetal species by culturing their cerebral cortex and blood in BSK II (Borrelia growth medium) [142]. In this study, the authors claim to have confirmed the presence of spirochetes in the blood, cerebral cortex, and CSF of 14 AD patients [129]. The bacterial isolates were further investigated by in situ hybridization and histopathology. These studies appeared to reveal that the spirochetes exist in neurofibrillary tangles and plaques of AD patients. In the process of interrogating Lyme disease patient autopsy specimens, our lab identified persisting *B. burgdorferi* in the CNS of a patient who was treated for Lyme disease and later developed Lewy body dementia [143]. This was supported by both molecular detection and specific antibody staining (immunofluorescence), revealing the spirochetes in the brain and spinal cord.

Nonetheless, several studies found no evidence to suggest that *B. burgdorferi* is linked to neurodegenerative disorders. Pappolla et al. tested post-mortem brain tissue samples from both AD cases and controls by culturing for Borrelia, but all were negative for growth [144]. In a similar approach, Gutaker et al. found no evidence of spirochetal infection in 10 postmortem AD brain samples, tested with standard and nested PCR, ELISA, and Western blotting for anti-*B. burgdorferi* antibodies [145]. Marques et al. also used PCR but did not detect *Borrelia* in any of the 30 postmortem AD and sex-matched control brain tissue samples [146]. Interestingly, McLaughlin et al. tested for spirochetes in the peripheral blood and fresh postmortem brain specimens of 22 patients with AD and 6 controls, and only 1 tested positive [147]. A large case-control study by Galbussera et al. found no evidence of *B. burgdorferi* in any serum samples using ELISA from 50 patients with AD, 23 controls, or 25 healthy caregivers of patients with AD [148]. In a nationwide population-based cohort study in Denmark, researchers observed no increased long-term risk of AD coincident with LD. This study is consistent with a geoepidemiology study from the US in which there were no associations between the geographical distribution of Lyme disease and the geographical distribution of AD [149]. It should be noted that Lyme disease is frequently associated with other tick- and non-tick-transmitted co-infections, suggesting that concurrent infections with several pathogens may also occur in Alzheimer’s disease. The most important of these co-infections are caused by *Bartonella* species, *Babesia* species, *Mycoplasma pneumoniae, Yersinia enterocolitica, C. pneumoniae,* HSV-1, and *C. trachomatis*. Co-infections with these pathogens may exacerbate Lyme disease symptoms through immune system modulation. Given the mixed findings, the etiologic role of *Borrelia burgdorferi* in the pathogenesis of AD remains unresolved.

### 2.4. Chlamydia pneumoniae

*Chlamydia pneumoniae* is an obligate, intracellular respiratory pathogen that can persist as a chronic infection for long periods of time. *C. pneumoniae* is responsible for a significant proportion of community-acquired pneumonia and has been linked to numerous other pulmonary diseases. Interestingly, *C. pneumoniae* has been associated with a range of non-respiratory diseases, including atherosclerosis, inflammatory arthritis, multiple sclerosis, and others [150,151,152]. Several authors reported an association between *C. pneumoniae* and AD since the bacteria has tropism for neural tissue. According to Shima and colleagues, *C. pneumoniae* could be a trigger for late-onset AD and is the most plausible of all infectious bacterial agents linked to AD pathogenesis [153]. In particular, the entry of the organism into the human brain is thought to occur following exposure in the respiratory tract. One potential route follows an intracellular infection of the neuroepithelia in the nasal airway to the olfactory bulb and then deeper into brain structures [154]. The other route would be following uptake in the lung by monocytes, which then traffic the organisms through the vasculature until they reach the brain through the BBB [154].

Immunohistochemical analyses of AD brains showed *C. pneumoniae* in various cell types found in the brain, including endothelial cells, astrocytes, microglia, and neurons [155,156,157,158,159,160,161]. The pathogen may reside in an intracellular inclusion/vacuole that resists immune recognition. These bacteria require cholesterol and sphingomyelin, which are gathered from the host; therefore, *C. pneumoniae* has the ability to manipulate the host cells [158]. It is proposed that *C. pneumoniae* and related antigens may interact with soluble oligomeric forms of amyloid in the same cortical regions of the brains of AD patients [1]. Currently, anti-chlamydia antibodies on the frontal and temporal cortical sections of AD brains are used to provide insight into the relationship between pathology and infection [158].

By employing a variety of techniques, including highly specific RT-PCR, immunoelectron microscopy, PCR, and in vivo cell culturing, researchers were able to localize infection in the AD brain. The areas found to be infected were the amygdala, entorhinal cortex, hippocampus proper, and the temporal and frontal cortices. Immunoelectron and electron microscopy identified chlamydial elementary bodies and reticulate bodies (RBs) [155,161]. The replicative RB form was detected in neurons, pericytes, and glial cells, which indicates that a viable and active form of *C. pneumoniae* is present in these cells [156,162,163,164]. In addition, Balin et al. found that 90% of AD brains, particularly the cerebral region, were found to be PCR-positive for the pathogen [155]. Schumacher et al. identified DNA of the organism in 90% of postmortem brain samples from LOAD patients and 5% of non-dementia control samples [20,150]. *C. pneumoniae* DNA was detected in the CSF of 43.9% of AD patients compared to 10.6% of the controls [165]. These findings suggest that *Chlamydia pneumoniae* infection in the brain should be considered a triggering agent in the initiation of AD pathogenesis. Other studies failed to detect *C. pneumoniae* DNA in the tissue of AD patients [20]. It should be noted that two of these studies were performed on paraffin-embedded tissues, which may have affected researchers’ ability to amplify DNA using the PCR technique [166,167].

### 2.5. Fungal Pathogens

Recently, researchers have advanced the idea that disseminated yeast and fungal infections contribute to the progression of AD. Under normal circumstances, *Candida* spp. live in the human intestinal tract along with other species of bacteria and yeast as a natural part of our microbial flora. *Candida albicans* is the most prolific cause of fungal infections in humans. The CDC estimates that roughly 25,000 cases of candidemia occur each year in the United States [168]. *C. albicans* is a commensal fungus that can readily infect organs and colonize inside resident cells and macrophages as endomycosomes or phagosomes [169]. The fungal AD hypothesis refers to the Carrasco et al. fungal etiology based on AD autopsies that associated fungi and neural tissue [170,171,172,173,174]. In the fungal model, a defeat of the innate immune system allows for the colonization of neural cells with fungi. Like a trojan horse, *Candida* spp. invade endothelial cells by endocytosis and escape the immune system by residing in endomycosomes. Fungi may enter into the cranial cavity via the nasopharyngeal nerve complex and then into the olfactory bulb, a region implicated in the development of dementia [175]. The fungi prevent removal by the immune system by maintaining low levels of colonization, which avoids activating apoptotic pathways [176]. As fungal burden increases, hyphal development induces cytokine production, which recruits neutrophils and macrophages. As the immune response clears the fungal burden, the colony falls from a virulent state to a low level of colonization [177]. Thus, researchers hypothesize that disseminated infection may slowly spread to the CNS, and neuronal loss takes place only when the fungal burden is high [170].

It has been suggested that Aβ functions as an antimicrobial peptide. Interestingly, *C. albicans* was found to be sensitive to synthetic Aβ and brain homogenates from AD patients that were capable of inhibiting fungal growth [178]. Additionally, it was demonstrated that Aβ protects against *C. albicans* in glial cells as well as in vivo in nematodes [179]. Therefore, the formation of Aβ is a response by the innate immune system in an attempt to protect against infection in the brain. Based on the assumption that fungi are the etiological agent of AD, all symptoms observed in the disease can readily be explained. The slow progression of Alzheimer’s disease reflects the chronic nature of fungal infections, if left untreated.

Alonso and colleagues provided extensive evidence that disseminated mycoses are potential causative agents or risk factors for AD [173,180]. Different fungal genera detected in AD brain tissue include *Malassezia, Fusarium, Candida, Cladosporium, Alternaria,* and *Botrytis* [181]. An analysis of CSF revealed significant levels of *Candida albicans* and *Candida glabrata* in samples from AD patients. Approximately 89.6% of serum from AD patients tested positive for antibodies to *Candida* compared to 8.8% for controls [173]. These studies revealed that many AD patients could have been co-infected with a variety of fungal species, while no fungal DNA was found in control samples. The different species that were detected included *Saccharomyces cerevisiae*, *Malassezia globosa*, *Malassezia restricta*, and *Penicillium*. Furthermore, this group detected yeast and fungal proteins, including (1,3)-β-glucan, fungal polysaccharides, and mycoses, in the peripheral blood of AD patients, which suggests that a chronic fungal infection may increase the risk of dementia [173,180]. More strikingly, yeast-like cells and hyphal structures were observed in CNS tissue from AD patients using polyclonal antibodies against a variety of fungi [181]. Pisa et al. also provided strong evidence for fungal infection in AD patients [170]. Brain sections derived from AD patients showed that all were infected with fungi [170]. Fungal material was detected intra- and extracellularly in the neurons of tissues of AD patients, but no fungal material was observed in tissue from control individuals [170]. Moreover, fungal DNA and proteins were also found in brain regions including the frontal cortex, hippocampus, cerebellar hemisphere, and choroid plexus but not in control patient tissue. Immunohistochemistry and confocal microscopy using anti-*C. glabrata* antibodies detected fungal bodies inside and outside nuclei and, in some cases, contained fungal nucleic acid [170]. The possibility that AD is a fungal disease opens new perspectives for treatment and therapy for patients. The aforementioned findings demonstrate that fungi can be detected in the CNS of AD patients and support the possibility that fungal infections are linked to AD.

## 3. Conclusions

This review highlights the knowledge gaps in the role of microbial pathogens as a risk factor for Alzheimer’s disease. A growing body of evidence supports that neuroinflammation is an essential component of AD pathology. Experimental and clinical data indicate the essential role of activation of the immune system in dementia progression. The persistent formation and deposition of amyloid-β peptide in the form of parenchymal plaques and abnormal aggregated tau protein in the form of NFT give rise to a chronic activation of the immune system. Interactions between activated glia and neurons, and possibly MAIT cells, around plaques maintain a chronic inflammatory state in the brain. Postmortem studies of AD brains suggest that infectious agents constitute a risk factor for AD. Research also suggests bacteria or viruses (e.g., HSV-1, *C. pneumoniae*, *C. albicans*, treponemal spp., and *B. burgdorferi*) can lead to cytokine dysregulation through a variety of mechanisms, including the activation of microglia and astrocytes, apoptosis, and altered neurotransmission. These effects accumulate over time and ultimately result in neurodegeneration. These effects, usually manifested during aging, can be exacerbated by several factors, including environmental risks, genetic alterations, and metabolic disorders. However, the infectious hypothesis of AD is still controversial, as no specific pathogen has been conclusively linked to the causation of AD in humans.

## 4. Future Directions

**Figure 6:**
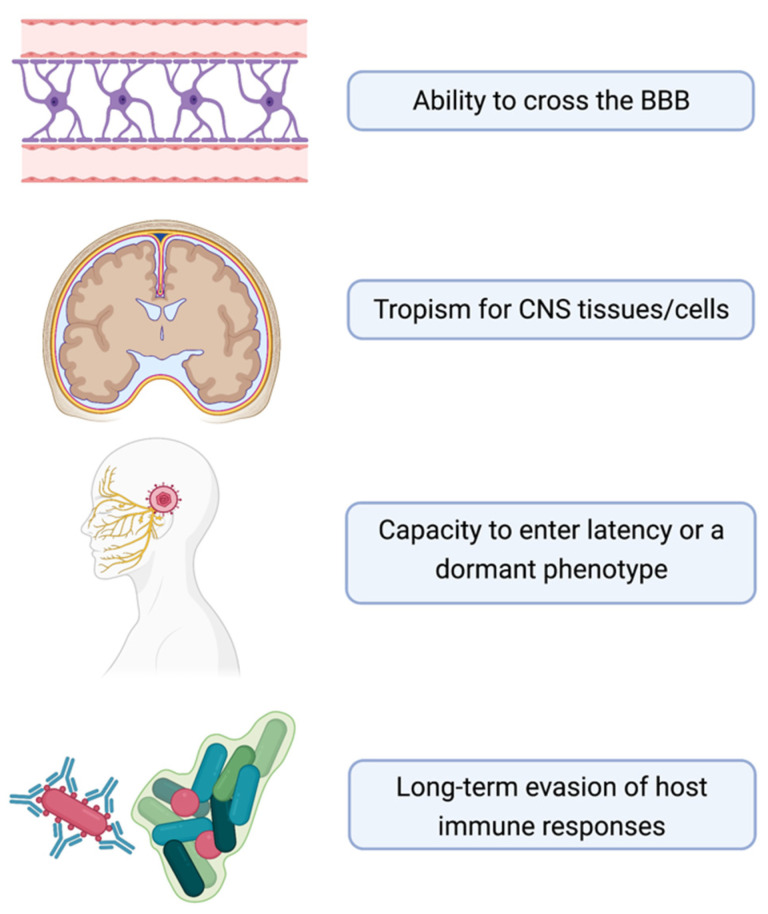
Common characteristics of pathogens implicated in the development of AD.

The data reviewed in this paper suggest that infectious agents can play a role in the pathogenesis of AD and related dementia and may provide opportunities for new preventative or therapeutic strategies aimed at counteracting the disease. We surmise that there are four common characteristics of pathogens that have been implicated in the development of AD (Figure 6). These include: (1) the ability to evade host immunity over the long term, possibly by inducing tolerance or encapsulation in protective biofilms; (2) the ability to cross the BBB, which may be more permeable in the elderly state; (3) tropism for the cells or tissues of the CNS; and (4) the ability to enter a latent or dormant phenotype, where the pathogen may be undetectable by standard clinical measures. The amyloid, inflammation, and microbial hypotheses have been described here. Both genetic and non-genetic factors appear to play a role in the development of AD. We propose that each of these hypotheses has merit, wherein either a genetic or microbial insult leads to immunosuppression, which allows the invasion or reactivation of a latent microbial infection, causing inflammation and amyloid production in the brain as a protective mechanism. If this cycle continues, neuronal damage ensues, leading to the clinical development of AD signs and symptoms. The availability of biomarker tests for the source of a (microbial) insult could lead to the more effective use of antimicrobials in the mitigation of the disease. In essence, should the evidence continue to mount in support of the microbial hypothesis, then the process could be halted with appropriate antimicrobials prior to excessive amyloid production, preventing disease.
